# The Effect of Music on Intraoperative Anxiety During Dermatologic Surgery: A Randomized Controlled Trial Comparing Rock and Classical Genres

**DOI:** 10.3390/bs16030317

**Published:** 2026-02-26

**Authors:** Alessandra Iorio, Francesca Sperati, Pasquale Frascione, Maria Perrone

**Affiliations:** 1 Department of Oncological and Preventative Dermatology, San Gallicano Dermatological Institute IRCCS, Istituti Fisioterapici Ospitalieri, 00144 Rome, Italy; pasquale.frascione@ifo.it; 2 UOSD Clinical Trial Center, Biostatistics and Bioinformatics, San Gallicano Dermatological Institute IRCCS, Istituti Fisioterapici Ospitalieri, 00144 Rome, Italy; francesca.sperati@ifo.it; 3 Psychology Unit, IRCCS “Regina Elena” National Cancer Institute, Via Elio Chianesi, 53, 00144 Rome, Italy; maria.perrone@ifo.it

**Keywords:** anxiety, dermatologic surgery, music, patient experience, randomized controlled trial, rock music, STAI

## Abstract

***Background:*** Music has been widely investigated as a non-pharmacological tool to reduce perioperative anxiety, yet its role in dermatologic surgery remains underexplored. This study aimed to evaluate the effect of different music genres on perioperative anxiety during awake dermosurgical procedures. ***Methods:*** We conducted a prospective, randomized, single-blind controlled trial at the San Gallicano Dermatological Institute IRCCS between March and December 2021. A total of 232 adult patients undergoing excision of atypical skin lesions were randomly assigned to three groups: rock music, classical music, or silence (control). Standardized playlists were played via speakers during surgery. Anxiety was assessed using the State-Trait Anxiety Inventory (STAI-Y1) at baseline (T0), immediately post-procedure (T1), and 48 h later (T2). Psychological profiles were further evaluated with the DASS-21 and patient-reported measures. ***Results:*** All groups showed a significant reduction in anxiety from T0 to T1 (*p* < 0.001). While between-group differences at T1 and T2 were not statistically significant, the greatest mean percentage reduction in anxiety was observed in the rock group (−16.8% ± 19.8), followed by classical (−15.7% ± 23.3) and silence (−11.6% ± 18.3). Patient satisfaction was highest in the rock group (9.3 ± 1.4; *p* = 0.015). Baseline psychological distress was higher among first-time surgical patients, especially in the rock group. ***Conclusions:*** Music, particularly rock, appears to be an effective adjunct to reduce anxiety and improve patient experience during awake dermatologic surgery. These findings support the integration of music into routine dermosurgical care.

## 1. Introduction

Dermo-oncological patients are particularly vulnerable; the initial diagnosis of skin cancer and the frequent identification of new or suspicious lesions can significantly increase anxiety and depression levels (Roberts et al., 2013; García-Montero et al., 2022; Radiotis et al., 2014). Surgery, whether a single occurrence or a repeated experience, can further exacerbate emotional distress and negatively impact well-being (Gogoularadja & Bakshi, 2020).

Unlike many other surgical settings where patients are sedated and primarily experience pre- and post-operative phases, dermatologic surgery is typically performed under local anesthesia. As a result, patients remain fully conscious, aware of visual, olfactory, and auditory stimuli throughout the procedure (Alam et al., 2016; Casale et al., 2018). Contributing factors to intraoperative discomfort include the unfamiliar surgical environment, restricted access for companions, and the painful administration of local anesthesia, often perceived as the most distressing aspect (Ilkkaya et al., 2014; Vaajoki et al., 2012; Riew et al., 2023; Arnold et al., 2024). The sensation of the surgical act itself, especially when it results in visible or permanent changes, may be experienced as a violation of bodily integrity (Gamboa et al., 2020).

These psychological responses are deeply individual, influenced by prior experiences, and may compromise patients’ quality of life if not adequately addressed (Nielsen et al., 2018; Gamboa et al., 2020). It is therefore essential to adopt strategies that enhance patient well-being, including clear communication, trust-building between patient and medical staff, and interventions that reduce sensory stressors.

Among such interventions, music has emerged as a simple yet effective tool to reduce anxiety and improve the perioperative experience. A growing body of evidence supports its anxiolytic, analgesic, and stress-mitigating effects during medical procedures (El Boghdady & Ewalds-Kvist, 2020; Gamboa et al., 2020; Lee et al., 2023; Stoneham et al., 2022; Vachiramon et al., 2013). Music engages brain regions associated with emotional regulation, memory, and attentional focus (Nilsson, 2008), acting as a positive distractor and promoting a sense of well-being (Gogoularadja & Bakshi, 2020; Casale et al., 2018).

While classical music has been traditionally favored in clinical settings, emerging data suggest that other genres, including rock music, may offer similar or even greater benefits depending on the context and individual preferences (Kupeli & Gülnahar, 2020; Arnold et al., 2024; Valevicius et al., 2023). However, well-designed studies directly comparing the effects of different music genres, particularly within the field of dermatologic surgery, remain scarce.

The present randomized study aims to assess whether listening to music (classical or rock) versus silence during dermatologic surgery can reduce intra- and post-operative anxiety and improve patient experience. It also seeks to evaluate patient satisfaction with different musical genres, thereby informing the development of targeted, genre-specific music interventions in dermatologic surgical practice.

## 2. Patients and Methods

### 2.1. Study Design and Setting

This study was a randomized, single-blind, controlled trial conducted at the Department of Oncological and Preventative Dermatology, San Gallicano Dermatological Institute IRCCS (a referral center for the treatment of dermatological diseases in Central Italy), between March and December 2021. The study was designed to address the following questions: (1) Does listening to music (classical or rock) during dermatologic surgery reduce intraoperative and post-operative anxiety compared to silence? (2) Are there differences in patient satisfaction between music genres (classical vs. rock)? (3) Does prior surgical experience influence psychological responses to music during awake dermatologic procedures? The study protocol adhered to the principles outlined in the Declaration of Helsinki and received approval from the Lazio Area 5 Ethics Committee (Ref. No. 1458/21, dated 23 February 2021).

### 2.2. Eligibility Criteria

Eligible participants were adults aged 18 to 90 years, with preserved auditory acuity and sufficient literacy to complete self-administered questionnaires. Inclusion required either a history of primary or multiple cutaneous melanoma and/or epitheliomas, or a clinical-dermoscopic diagnosis of atypical lesions, with no prior history of major comorbidities.

Exclusion criteria included a documented history of psychiatric illness or any evidence of conditions that could interfere with study participation.

### 2.3. Procedures

Following informed consent for both the surgical procedure and study participation, patients underwent dermatologic surgery for the excision of atypical lesions. A randomized controlled design was implemented, with block randomization by surgical day. Randomization was performed using a block randomization scheme by surgical day to ensure balanced group allocation. The randomization sequence was computer-generated by a study investigator who was not involved in patient recruitment or clinical care. Block sizes varied from 6 to 9 participants per surgical day. Allocation concealment was ensured using sealed, opaque envelopes that were opened immediately before the surgical procedure. All surgeries were performed by the same dermo-surgeon, assisted by a single nurse, and interactions with the surgical team were standardized to minimize potential external influences on patient experience.

Participants were randomly allocated on the day of surgery to one of three predefined groups: (1) rock music, (2) classical music, or (3) no music (control). The assignment followed a day-based randomization schedule, with all patients treated on the same day exposed to the same genre. Within each procedure, tracks were presented in a randomized sequence that was not repeated across sessions. For the music intervention groups, two standardized playlists, one for classical and one for rock music, were created based on general musical knowledge and familiarity, without aiming for exhaustive representation. Each playlist contained approximately 35–45 tracks. Music was played aloud via portable wireless Bluetooth speakers connected to a playback device, maintaining a consistent volume between 50 dB and 60 dB. We selected two clearly contrasting and representative musical genres—classical and rock music—to maximize discriminative power at this exploratory stage.

Music genre allocation was randomized and independent of the patient’s musical preferences, which were not disclosed in advance. This approach was adopted to facilitate an unbiased evaluation of the emotional and perceptual responses elicited by the different music genres.

The rock playlist included tracks by artists such as AC/DC, Offspring, Stereophonics, Metallica, Dire Straits, Kiss, Rolling Stones, Queen, The Killers, Guns N’ Roses, Aerosmith, Vasco Rossi, Oasis and Linkin Park. The classical music playlist featured compositions by Pyotr Ilyich Tchaikovsky, Frédéric Chopin, Antonio Vivaldi, Ludovico Einaudi, Johann Strauss, Sergei Rachmaninoff, and Antonín Dvořák (see the full list in the Supplementary Materials). Music was played continuously throughout the surgical procedure, with the duration matched to the length of the operation.

In the control group, the surgical team conducted the procedure in silence, limiting communication to essential postoperative instructions regarding scar care.

### 2.4. Psychological Measures and Outcome Variables

This study assessed changes in patients’ psychological well-being before and after dermatologic surgery, in relation to the presence and type of musical intervention. Additional psychological dimensions were evaluated, including patients’ emotional state in the week prior to surgery, their understanding of the clinical diagnosis, and satisfaction with the information received.

Data were collected at four defined time points. At baseline (T0, pre-operative), patients completed the State-Trait Anxiety Inventory (STAI), which includes 40 self-administered items to assess both state anxiety (form Y-1) and trait anxiety (form Y-2), with scores ranging from 20 to 80 (higher scores indicate greater anxiety) (Ilya et al., 2025). They also completed the Depression Anxiety Stress Scales-21 (DASS-21), a 21-item questionnaire that evaluates depression, anxiety, and stress. Three additional visual analog scale questions assessed self-perceived emotional status over the previous week (Question 1), understanding of the clinical prognosis (Question 2), and satisfaction with the information provided (Question 3). Each was scored from 1 to 10, with higher scores reflecting more favorable perceptions. Baseline psychological profiles were included to assess group comparability and to explore potential moderating effects (e.g., first-time surgery status).

Immediately following surgery (T1), patients repeated the STAI Y-1 to reassess state anxiety and responded to additional satisfaction-related questions. Those in the music groups rated their satisfaction with the musical genre (Question 4a) and its perceived effect on emotional stress (Question 5), while participants in the silence group rated their comfort during the procedure (Question 4b), again using a 1–10 scale.

A final assessment was conducted 48 h post-surgery (T2), during which patients completed the STAI Y-1 at home. The completed questionnaires were returned during the scheduled visit for suture removal and histological result disclosure. All instruments were self-administered to minimize interpersonal bias and enhance data consistency.

### 2.5. Statistical Analysis

Categorical variables were reported as absolute and relative frequencies, while continuous variables were summarized using means, standard deviations (SD), medians, and minimum–maximum ranges. The Kolmogorov–Smirnov test was used to assess the normality of distribution for all continuous variables. Between-group differences in continuous variables were analyzed using the Kruskal–Wallis test or the Mann–Whitney U test, as appropriate according to the Kolmogorov–Smirnov test. To account for multiple comparisons, Bonferroni correction was applied (adjusted significance threshold: *p* ≤ 0.017, based on 0.05/3). To evaluate within-subject changes over time—specifically between baseline (T0) and immediate post-operative (T1), and between baseline and 48 h post-surgery (T2)—the Wilcoxon signed-rank test was used. Delta values were calculated as the relative percentage change from baseline: the difference between follow-up and baseline scores was divided by the baseline value and multiplied by 100.

In addition to individual group comparisons, a pooled analysis was conducted by aggregating the two music groups (rock and classical) and comparing them against the silence group. This allowed us to assess the general effect of music, regardless of genre, on anxiety outcomes. Differences in anxiety scores (T0–T1 and T0–T2) were evaluated using the Mann–Whitney U test, and percentage changes were compared to identify the magnitude of the music intervention effect. A subgroup exploratory post hoc analysis was conducted to compare psychological outcomes between patients undergoing their first dermatosurgical procedure and those with prior surgical experience. This comparison was stratified by intervention group (silence, classical, rock) and included baseline and follow-up assessments of anxiety (STAI-Y1) and perceived stress. The Wilcoxon signed-rank test was used to evaluate within-group changes from T0 to T1. All statistical analyses were conducted using SPSS software, version 29 (IBM SPSS Statistics, Chicago, IL, USA). A *p*-value of <0.05 was considered statistically significant unless otherwise adjusted for multiple testing.

## 3. Results

### 3.1. Study Population and Group Allocation

A total of 232 patients were enrolled and underwent dermatologic surgery for the excision of atypical lesions. Participants were randomly assigned to one of three groups: 79 to the silence group, 73 to the classical music group, and 80 to the rock music group.

As shown in Table 1, the three groups were comparable in terms of key demographic and clinical variables. The mean age was similar across groups (49.8 ± 14.9 in the silence group, 51.9 ± 12.1 in the classical group, and 49.3 ± 14.1 in the rock group; *p* = 0.470). Gender distribution was also balanced, with approximately equal proportions of male and female patients (*p* = 0.739).

Educational attainment did not differ significantly across groups (*p* = 0.212), with the majority holding either a secondary school diploma or a university degree. Similarly, no significant differences were found in histological diagnoses (*p* = 0.228), with melanomas, especially pT1a, being the most common malignant lesions, followed by non-pathological findings and epithelial tumors.

Psychological characteristics at baseline (T0) were also similar across groups (Table 2). Anxiety levels assessed using the DASS-21 were 2.8 ± 3.5 in the silence group, 2.7 ± 2.8 in the classical music group, and 2.7 ± 3.0 in the rock music group (*p* = 0.739). Depression scores were 2.4 ± 3.1, 2.9 ± 3.1, and 3.0 ± 3.2, respectively (*p* = 0.301), while stress scores were 4.9 ± 3.8, 5.5 ± 3.7, and 5.4 ± 4.1 (*p* = 0.492). Patient-reported VAS scores for perceived understanding of prognosis (8.2 ± 2.0, 7.9 ± 2.0, and 8.0 ± 1.9; *p* = 0.508) and satisfaction with information received (8.2 ± 2.0, 8.5 ± 1.4, and 8.2 ± 1.9; *p* = 0.899) were also comparable among the three groups. However, a significant difference emerged in the self-reported emotional state over the previous week. Patients in the rock music group reported lower VAS scores (6.1 ± 2.3) compared to those in the classical (6.8 ± 2.1) and silence (7.0 ± 2.2) groups (*p* = 0.044), although post hoc comparisons did not show significant differences between individual groups.

Additionally, within the rock group, patients undergoing surgery for the first time exhibited significantly higher levels of anxiety (*p* = 0.025) and depression (*p* = 0.011) compared to those with previous surgical experience (Table S1). No such differences were found in the classical music or silence groups (Tables S2 and S3).

### 3.2. Effect of Music on Pre- and Post-Operative Anxiety (T0–T1)

Table 3 depicts STAI-Y1 and subjective assessments at T0 and T1 by intervention group. Baseline anxiety levels were comparable across groups (*p* = 0.815). At T1, no significant differences in STAI-Y1 scores were observed between groups (*p* = 0.545). However, within-group comparisons revealed a significant reduction in anxiety from T0 to T1 in all three groups (*p* < 0.001 for each), indicating a general decrease in state anxiety following the procedure.

The analysis of percentage change in anxiety (Δ T0–T1) showed a greater reduction in the music groups compared to the silence group. The rock music group exhibited the largest mean reduction (−16.8% ± 19.8%), followed by the classical music group (−15.7% ± 23.3%) and the silence group (−11.6% ± 18.3%), although the between-group difference did not reach statistical significance (*p* = 0.081).

Satisfaction with the auditory environment at T1 differed significantly between groups (*p* = 0.015), with the highest ratings in the rock music group (mean 9.3 ± 1.4), followed by classical music (8.6 ± 2.0) and silence (8.5 ± 2.3). Post hoc analysis with Bonferroni correction confirmed significantly higher satisfaction in the rock group compared to both silence (*p* = 0.015) and classical music (*p* = 0.010).

Among participants in the music groups, the perceived impact of music on stress did not differ significantly between genres (*p* = 0.340).

### 3.3. Effects at 48 Hours Post-Surgery (T0–T2)

The number and proportion of patients lost to follow-up were comparable across groups: 59 out of 80 patients (73.8%) in the rock music group, 53 out of 73 (72.6%) in the classical music group, and 53 out of 79 (67.1%) in the silence group returned the STAI-Y1 questionnaire. In the subgroup of patients who completed follow-up assessments at T2, anxiety levels at baseline were similar across the three groups: mean STAI-Y1 scores were 35.4 ± 10.4 in the silence group (*n* = 53), 37.9 ± 10.1 in the classical music group (*n* = 53), and 37.5 ± 10.6 in the rock music group (*n* = 59), with no statistically significant differences (*p* = 0.339) (Table 4).

At T2, anxiety levels remained comparable between groups, with mean STAI-Y1 scores of 33.2 ± 9.5 in the silence group, 34.2 ± 9.2 in the classical music group, and 32.4 ± 9.0 in the rock music group (*p* = 0.410).

Within-group comparisons showed a statistically significant reduction in anxiety from T0 to T2 in both the rock music group (mean reduction: −5.1 points; *p* = 0.001) and the classical music group (mean reduction: −3.7 points; *p* = 0.004). In contrast, the silence group showed a smaller, non-significant decrease (mean reduction: −2.2 points; *p* = 0.067).

Analysis of percentage change in anxiety (Δ T0–T2) confirmed a greater reduction in the music groups: the rock group showed a mean decrease of −7.9% ± 33.9, and the classical group −7.6% ± 18.7, compared to −3.8% ± 20.1 in the silence group. However, the between-group difference in percentage change did not reach statistical significance (*p* = 0.173).

### 3.4. Pooled Music vs. Silence Comparisons

As shown in Table 5, baseline anxiety levels were comparable between the silence group (mean 36.9 ± 11.0) and the music group (mean 37.6 ± 10.4), with no statistically significant difference (*p* = 0.565). Similarly, anxiety levels immediately after surgery (T1) remained statistically similar between the two groups: 31.9 ± 10.0 in the silence group versus 30.3 ± 8.2 in the music group (*p* = 0.282).

However, analysis of the percentage change in anxiety from T0 to T1 (Δ T0–T1) revealed a significantly greater reduction in the music group (mean −16.3% ± 21.5) compared to the silence group (mean −11.6% ± 18.3; *p* = 0.026), indicating a more pronounced anxiolytic effect associated with music exposure during surgery. Music preference at T1 did not differ significantly between genres (*p* = 0.178).

In the subsample of patients with complete follow-up at 48 h (T2), baseline anxiety levels were slightly lower in the silence group (mean 35.4 ± 10.4) compared to the music group (mean 37.7 ± 10.3), but the difference was not statistically significant (*p* = 0.154) (Table 6). By T2, anxiety levels were nearly identical across both groups, with mean STAI-Y1 scores of 33.2 ± 9.5 in the silence group and 33.2 ± 9.1 in the music group (*p* = 0.973). Within-group analysis showed a statistically significant reduction in anxiety from T0 to T2 in the music group (*p* < 0.001), while the reduction in the silence group did not reach significance (*p* = 0.067). The percentage reduction in anxiety (Δ T0–T2) was greater in the music group (mean −7.8% ± 27.6) compared to the silence group (mean −3.8% ± 20.1), although the difference between groups was not statistically significant (*p* = 0.131).

### 3.5. First-Time vs. Experienced Patients

A supplementary subgroup analysis was conducted to compare anxiety and stress outcomes between patients undergoing their first dermatosurgical procedure and those with prior surgical experience. The aim was to assess whether procedural familiarity moderated emotional responses across the three intervention groups. Full results are presented in Table 7.

At baseline (T0), patients undergoing their first surgery generally reported higher anxiety scores compared to experienced patients, particularly in the classical music group (mean STAI-Y1: 40.4 ± 10.1 vs. 36.7 ± 9.8). Despite this, all subgroups exhibited statistically significant reductions in anxiety immediately after surgery (T1), with *p* < 0.05 in every comparison.

The rock music group showed the most consistent reductions in both first-time and experienced patients (T0–T1 STAI-Y1 reductions: 36.7 ± 8.9 to 30.0 ± 5.5 in first-timers; 37.9 ± 11.5 to 29.8 ± 7.8 in experienced), accompanied by a significant increase in perceived stress relief (stress VAS rising from 6.0 to 6.1 at T0 to 9.1 at T1; *p* < 0.001 for both). A similar pattern was observed in the classical group, though first-time patients started with higher anxiety and ended with higher residual anxiety post-operatively (T1: 34.4 ± 12.7 vs. 29.6 ± 7.7).

In the silence group, stress outcomes were not available, but anxiety reductions were also observed in both first-time and experienced patients.

## 4. Discussion

This study suggests that music, particularly rock music, may enhance the dermosurgical experience by reducing intraoperative anxiety. While between-group differences were not always statistically significant, a consistent trend favored music over silence, with rock music emerging as both the most effective and most appreciated genre. This challenges the traditional clinical preference for classical music (Casale et al., 2018; Kupeli & Gülnahar, 2020).

The anxiolytic effects observed are consistent with previous findings in surgical settings, where music has been shown to reduce discomfort and anxiety (Gogoularadja & Bakshi, 2020; Nilsson, 2008). In dermosurgery, where patients remain awake and exposed to potentially distressing stimuli, music may act as a sensory buffer and cognitive distractor, masking unfamiliar sounds and shifting attention toward more positive cues (Casale et al., 2018; Nielsen et al., 2018).

To minimize bias, participants were randomized to predefined playlists based on general familiarity rather than personal preference. Despite this, rock music was not only better received but also associated with the greatest anxiety reduction—contrary to the expectation that classical music is universally calming (Kupeli & Gülnahar, 2020). Patients in the rock group reported lower self-rated emotional states at baseline (VAS scores). However, no significant differences in baseline anxiety or depression were observed between groups according to DASS-21 or STAI-Y1 measures (Table 2 and Table 7). Therefore, while this may suggest greater preoperative emotional tension, the available data do not allow definitive conclusions regarding greater psychological vulnerability in this group. Further research would be needed to explore this hypothesis. Anxiety reductions persisted 48 h after surgery, particularly in music groups, supporting the idea of a residual anxiolytic effect. However, missing data from incomplete questionnaires or unreturned follow-ups may have limited the power of some subgroup analyses. Moreover, the influence of post-surgical recovery or other contextual factors cannot be excluded. Accordingly, no definitive conclusions can be drawn from these data.

The consistent preference for rock music—rarely used in healthcare settings (Kupeli & Gülnahar, 2020; Oomens et al., 2019; Kühlmann et al., 2018; Casale et al., 2018)—highlights its potential to create a more engaging and supportive environment in conscious surgical procedures. These results align with recent studies showing music’s ability to reduce perioperative stress and improve patient experience (Giordano et al., 2023; Goel et al., 2024).

This study also explored whether previous surgical experience influenced patients’ psychological responses to music. The subgroup analysis showed that patients undergoing their first surgical procedure tended to report higher baseline anxiety across groups, particularly in the classical music arm. Despite these differences, all subgroups experienced significant reductions in anxiety after surgery, supporting the robustness of the music intervention. Notably, rock music was associated with consistently favorable outcomes regardless of surgical experience, suggesting its broad applicability in reducing intraoperative stress. These findings highlight the importance of considering procedural familiarity when evaluating patient-centered interventions in surgical settings.

Several limitations must be acknowledged. Emotional variability in dermo-oncological patients was not fully controlled and may have influenced psychological outcomes. Procedural stressors such as being awake during surgery, exposure to unfamiliar environments, and local anesthesia could have amplified anxiety and obscured the full impact of the music. Music delivered via speakers may have influenced staff behavior or the perceived environment. Additionally, the silence condition may not have been entirely silent. These contextual factors represent potential limitations. Moreover, brief interactions with the surgical team may have introduced interpersonal influences. Geographic, cultural, and socioeconomic differences could also limit generalizability. Although the use of standardized playlists allowed for control over genre exposure and enhanced reproducibility, this approach may have limited the individual emotional resonance of the intervention. Several studies, including recent work by Arnold et al. (2024), suggest that patient-selected music—closely aligned with personal preferences—may elicit stronger psychophysiological responses and produce greater reductions in anxiety and pain. While our design prioritized methodological consistency, future studies should assess whether allowing patients to choose their preferred music could yield superior and more consistent outcomes in dermosurgical care. Similarly, incorporating qualitative data, such as interviews or free-text feedback, could offer deeper insight into how patients experience music in surgical settings. Lastly, the potential for a Hawthorne effect must be considered: patients may have altered their behavior due to study participation alone, independent of the intervention.

Despite these limitations, the findings support the use of music as a complementary strategy to improve patient well-being during dermosurgery. The unexpected preference for rock music, alongside its anxiolytic potential, suggests that high-energy music may be especially suitable in this context. Moreover, music may foster a sense of connection and trust between patients and surgical staff, reinforcing its broader therapeutic value (El Boghdady & Ewalds-Kvist, 2020; Riew et al., 2023; Valevicius et al., 2023; Casale et al., 2018; Kupeli & Gülnahar, 2020).

## 5. Conclusions

These findings offer a valuable foundation for future research aimed at systematically exploring a wider spectrum of musical genres, assessing the impact of patient-selected music, and incorporating qualitative insights into the emotional experiences of patients undergoing awake procedures. Such investigations may deepen our understanding of how specific musical characteristics influence psychological responses, ultimately guiding the development of tailored, non-pharmacological interventions to enhance perioperative well-being in oncologic care. Looking forward, the integration of music into dermatologic surgical practice emerges as a simple, safe, and cost-effective strategy with the potential to reduce anxiety and stress and enrich the overall patient experience.

## Figures and Tables

**Table 1:** Demographic and Clinical Characteristics by Intervention Group.

Variable	Silence (*n* = 79), *n* (%)	Classic (*n* = 73)	Rock (*n* = 80)	*p*-Value
Age (years), mean ± SD	49.8 ± 14.9	51.9 ± 12.1	49.3 ± 14.1 ^#^	0.470 *
Gender				0.739 **
Female	41 (51.9%)	41 (56.2%)	40 (50.0%)	
Male	38 (48.1%)	32 (43.8%)	40 (50.0%)	
Educational level ^§^				0.212 **
Primary school	1 (1.1%)	1 (1.1%)	1 (1.1%)	
Secondary School Diploma	43 (49.4%)	30 (34.5%)	33 (37.5%)	
University degree	43 (49.4%)	56 (64.4%)	52 (59.1%)	
Histological diagnosis				0.228 **
Clark con severe atypia	1 (1.1%)	1 (1.1%)	1 (1.1%)	
Epithelial carcinoma	20 (23.0%)	8 (9.2%)	1 (12.5%)	
Non pathological lesion	33 (37.9%)	29 (33.3%)	35 (39.8%)	
Melanoma in situ	10 (11.5%)	11 (12.6%)	14 (15.9%)	
Melanoma pT1a	22 (25.3%)	37 (42.5%)	25 (28.4%)	
Melanoma pT1b	0 (0.0%)	0 (0.0%)	1 (1.1%)	
Melanoma pT2a	0 (0.0%)	0 (0.0%)	1 (1.1%)	
Metastasis melanoma	0 (0.0%)	1 (1.1%)	0 (0.0%)	
Negative	1 (1.1%)	0 (0.0%)	0 (0.0%)	

* ANOVA one-way test. ** Chi2 test. ^#^ 1 missing. ^§^ 2 missing in the rock music group.

**Table 2:** Baseline Psychological Characteristics by Intervention Group.

Variable	Silence (*n* = 79)	Classical Music (*n* = 73)	Rock Music (*n* = 80)	*p*-Value ^†^
DASS-21 Anxiety				0.739
Mean ± SD	2.8 ± 3.5	2.7 ± 2.8	2.7 ± 3.0	
Median (Min–Max)	2 (0–20)	2 (0–13)	2 (0–14)	
DASS-21 Depression				0.301
Mean ± SD	2.4 ± 3.1	2.9 ± 3.1	3.0 ± 3.2	
Median (Min–Max)	1 (0–14)	2 (0–16)	2 (0–15)	
DASS-21 Stress				0.492
Mean ± SD	4.9 ± 3.8	5.5 ± 3.7	5.4 ± 4.1	
Median (Min–Max)	4 (0–21)	5 (0–16)	5 (0–19)	
VAS: Emotional State (past week)				0.044
Mean ± SD	7.0 ± 2.2	6.8 ± 2.1	6.1 ± 2.3	
Median (Min–Max)	7 (1–10)	7 (1–10)	6 (1–10)	
VAS: Understanding of Prognosis				0.508
Mean ± SD	8.2 ± 2.0	7.9 ± 2.0	8.0 ± 1.9	
Median (Min–Max)	9 (2–10)	8 (1–10)	8 (3–10)	
VAS: Satisfaction with Information				0.899
Mean ± SD	8.2 ± 2.0	8.5 ± 1.4	8.2 ± 1.9	
Median (Min–Max)	9 (2–10)	9 (5–10)	9 (3–10)	

^†^ Kruskal–Wallis test. Post hoc pairwise comparisons for emotional state (VAS) were not statistically significant.

**Table 3:** State Anxiety (STAI-Y1) and Subjective Assessments at T0 and T1 by Intervention Group.

Variable	Silence (*n* = 79)	Classical Music (*n* = 73)	Rock Music (*n* = 80)	*p*-Value
STAI-Y1 (T0)				0.815 ^†^
Mean ± SD	36.9 ± 11.0	37.6 ± 10.0	37.6 ± 10.8	
Median (Min–Max)	35.0 (20.0–66.0)	36.0 (18.0–65.0)	35.0 (17.0–64.0)	
STAI-Y1 (T1)				0.545 ^†^
Mean ± SD	31.9 ± 10.0	30.7 ± 9.2	29.9 ± 7.2	
Median (Min–Max)	31.0 (20.0–67.0)	30.0 (20.0–69.0)	29.0 (20.0–59.0)	
Within-group change (T0 vs. T1)				
Wilcoxon signed-rank test	<0.001	<0.001	<0.001	
Δ STAI-Y1 T0–T1 (% change)				0.081 ^†^
Mean ± SD	−11.6 ± 18.3	−15.7 ± 23.3	−16.8 ± 19.8	
Median (Min–Max)	−10.2 (−61.5 to 70.0)	−17.2 (−53.8 to 81.6)	−14.1 (−63.6 to 48.4)	
Satisfaction with intervention (T1)				0.015 ^‡^
Mean ± SD	8.5 ± 2.3	8.6 ± 2.0	9.3 ± 1.4	
Median (Min–Max)	10.0 (1.0–10.0)	9.0 (1.0–10.0)	10.0 (4.0–10.0)	
Perceived impact on stress (T1)	–	9.0 ± 1.7	9.1 ± 1.8	0.340 ^§^
Median (Min–Max)	–	10.0 (1.0–10.0)	10.0 (1.0–10.0)	

^†^ Kruskal–Wallis test. ^‡^ Kruskal–Wallis test with Bonferroni correction: silence vs. rock, *p* = 0.015; classical vs. rock, *p* = 0.010. ^§^ Mann–Whitney U test (music groups only).

**Table 4:** State Anxiety (STAI-Y1) at T0 and T2 in Patients with Complete Follow-Up.

Variable	Silence (*n* = 53)	Classical Music (*n* = 53)	Rock Music (*n* = 59)	*p*-Value ^†^
STAI-Y1 (T0)				0.339
Mean ± SD	35.4 ± 10.4	37.9 ± 10.1	37.5 ± 10.6	
Median (Min–Max)	34.0 (20.0–61.0)	36.0 (20.0–65.0)	35.0 (17.0–64.0)	
STAI-Y1 (T2)				0.410
Mean ± SD	33.2 ± 9.5	34.2 ± 9.2	32.4 ± 9.0	
Median (Min–Max)	31.0 (20.0–61.0)	31.0 (20.0–60.0)	30.0 (20.0–61.0)	
Within-group change (T0 vs. T2)				
Wilcoxon signed-rank test	0.067	0.004	0.001	
Δ STAI-Y1 T0–T2 (% change)				0.173
Mean ± SD	−3.8 ± 20.1	−7.6 ± 18.7	−7.9 ± 33.9	
Median (Min–Max)	−4.8 (−48.1 to 45.0)	−5.3 (−53.8 to 37.1)	−12.5 (−64.1 to 147.1)	

^†^ Kruskal–Wallis test.

**Table 5:** Comparison of Anxiety and Satisfaction Between Silence and Music Groups at T0 and T1.

Variable	Silence (*n* = 79)	Music (Classical + Rock) (*n* = 153)	*p*-Value ^†^
STAI-Y1 (T0)			0.565
Mean ± SD	36.9 ± 11.0	37.6 ± 10.4	
Median (Min–Max)	35.0 (20.0–66.0)	35.0 (17.0–65.0)	
STAI-Y1 (T1)			0.282
Mean ± SD	31.9 ± 10.0	30.3 ± 8.2	
Median (Min–Max)	31.0 (20.0–67.0)	29.0 (20.0–69.0)	
Within-group change (T0 vs. T1)			
Wilcoxon signed-rank test	<0.001	<0.001	–
Δ STAI-Y1 T0–T1 (% change)			0.026
Mean ± SD	−11.6 ± 18.3	−16.3 ± 21.5	
Median (Min–Max)	−10.2 (−61.5 to 70.0)	−17.1 (−63.6 to 81.6)	
Satisfaction with auditory environment (T1)			0.178
Mean ± SD	8.5 ± 2.3	9.0 ± 1.8	
Median (Min–Max)	10.0 (1.0–10.0)	10.0 (1.0–10.0)	

^†^ Mann–Whitney U test.

**Table 6:** Comparison of Anxiety Between Silence and Music Groups at T0 and T2 (Subsample with Complete Follow-Up).

Variable	Silence (*n* = 53)	Music (Classical + Rock) (*n* = 112)	*p*-Value ^†^
STAI-Y1 (T0)			0.154
Mean ± SD	35.4 ± 10.4	37.7 ± 10.3	
Median (Min–Max)	34.0 (20.0–61.0)	35.0 (17.0–65.0)	
STAI-Y1 (T2)			0.973
Mean ± SD	33.2 ± 9.5	33.2 ± 9.1	
Median (Min–Max)	31.0 (20.0–61.0)	31.0 (20.0–61.0)	
Within-group change (T0 vs. T2)			
Wilcoxon signed-rank test	0.067	<0.001	–
Δ STAI-Y1 T0–T2 (% change)			0.131
Mean ± SD	−3.8 ± 20.1	−7.8 ± 27.6	
Median (Min–Max)	−4.8 (−48.1 to 45.0)	−9.4 (−64.1 to 147.1)	

^†^ Mann–Whitney U test.

**Table 7:** Anxiety and Stress Levels by Surgery Experience and Intervention Group.

Group	First Surgery	*n*	STAI-Y1 Baseline (Mean ± SD)	STAI-Y1 T1 (Mean ± SD)	Wilcoxon Test (T0–T1)	Stress T0 (Mean ± SD)	Stress T1 (Mean ± SD)	Wilcoxon Test (Stress)
Silence	No	53	35.9 ± 10.9	31.8 ± 9.8	<0.001	7.0 ± 2.3	n.a. *	n.a.
Silence	Yes	20	38.8 ± 11.2	32.1 ± 12.1	0.001	7.2 ± 1.6	n.a.	n.a.
Rock	No	59	37.9 ± 11.5	29.8 ± 7.8	<0.001	6.0 ± 2.4	9.1 ± 1.9	<0.001
Rock	Yes	20	36.7 ± 8.9	30.0 ± 5.5	<0.001	6.1 ± 2.2	9.1 ± 1.6	<0.001
Classical	No	56	36.7 ± 9.8	29.6 ± 7.7	<0.001	6.9 ± 1.8	9.2 ± 1.5	<0.001
Classical	Yes	17	40.4 ± 10.1	34.4 ± 12.7	0.039	6.3 ± 2.8	8.6 ± 2.3	0.009

* n.a. = not available.

## Data Availability

The patient data used in this study are stored in a secure internal database and cannot be made publicly available due to privacy regulations and ethical restrictions. Anonymized data may be available from the corresponding author upon reasonable request and with appropriate ethical approval.

## Supplementary Materials

The following supporting information can be downloaded at: https://www.mdpi.com/article/10.3390/bs16030317/s1, Table S1: Baseline Psychological Characteristics in the Rock Music Group According to Surgical Experience; Table S2: Baseline Psychological Characteristics in the Classical Music Group According to Surgical Experience; Table S3. Baseline Psychological Characteristics in the Silence Group According to Surgical Experience.
