# Supplementary material for: The Effect of Music on Intraoperative Anxiety During Dermatologic Surgery: A Randomized Controlled Trial Comparing Rock and Classical Genres
Source: Behav Sci (Basel). 2026 Feb 26;16(3):317. doi: 10.3390/bs16030317 (PMC13024541; doi:10.3390/bs16030317)
Supplement: Supplementary file 1 [file behavsci-16-00317-s001.zip › behavsci-4080370-supplementary.pdf]

SUPPLEMENTARY MATERIAL

Music playlists used in the study

Rock music playlist

- The rock music playlist included widely recognizable and commercially popular tracks selected to ensure familiarity across a broad adult population. The following artists and tracks were included:
- AC/DC: Highway to Hell; Back in Black; Anything Goes; Thunderstruck; Moneytalks; Rock 'n' Roll Train.
- The Offspring: Pretty Fly (For a White Guy); The Kids Aren't Alright; Why Don't You Get a Job.
- The Killers: Mr. Brightside; Human; Read My Mind; Somebody Told Me.
- Kiss: Rock and Roll All Nite; I Was Made for Lovin' You; Hard Luck Woman; Detroit Rock City.
- Queen: Bohemian Rhapsody; A Little Thing Called Love; I Want to Break Free; I Want It All; Don't Stop Me Now; We Will Rock You; Hammer to Fall.
- The Doors: Roadhouse Blues; Light My Fire.
- Guns N' Roses: Knockin' on Heaven's Door; Live and Let Die; Welcome to the Jungle; Paradise City; Sweet Child O' Mine; November Rain.
- Metallica: Enter Sandman; Fade to Black; One.
- Aerosmith: Jaded; Dream On; Pink; Rag Doll; Cryin'; Crazy; Walk This Way; Hole in My Soul.
- Van Halen: Jump.
- The Cranberries: Zombie.
- The White Stripes: Seven Nation Army.
- Dire Straits: Walk of Life; Sultans of Swing; Two Young Lovers.
- Blur: Song 2.
- Linkin Park: In the End; Numb; Burn It Down; Somewhere I Belong.
- Oasis: The Shock of the Lightning; Supersonic; Rock 'N' Roll Star; Cigarettes & Alcohol; Slide Away; Acquiescence; Wonderwall.
- Stereophonics: Dakota; Do Ya Feel My Love?; The Bartender and the Thief; Caught by the Wind; Pick a Part That's New.
- Vasco Rossi: Rewind; C'è Chi Dice No; Gioca con Me; Gli Spari Sopra; Ti Prendo e Ti Porto Via; Basta Poco.

Classical music playlist

The classical music playlist consisted of well-known orchestral and piano works commonly used in relaxing and emotionally engaging listening contexts:

- Pyotr Ilyich Tchaikovsky: orchestral selections from Swan Lake, The Nutcracker, and Sleeping Beauty.
- Antonio Vivaldi: The Four Seasons (all four concertos).
- Frédéric Chopin: Étude Op. 10 No. 3; Nocturne Op. 9 No. 1; Nocturne Op. 9 No. 2; Nocturne No. 20 in C-sharp minor; Prelude Op. 28 No. 4; Piano Sonata No. 2; Fantaisie-Impromptu.
- Johann Strauss II: Voices of Spring; On the Beautiful Blue Danube; Vienna Blood; Roses from the South; Artist's Life; Tales from the Vienna Woods.
- Sergei Rachmaninov: Piano Concerto No. 3.
- Antonín Dvořák: Serenade for Strings in E major.

Table S1. Baseline Psychological Characteristics in the Rock Music Group According to Surgical Experience

**Variable**	**Previous surgery (n=59)**	**First surgery (n=20)**	**Mann–Whitney U test (p-value)**
DASS-21 anxiety			**0.025**
• Mean ± SD	3.1 ± 3.1	1.7 ± 2.4	
• Median (Min–Max)	2 (0–14)	0.5 (0–8)	
DASS-21 depression			**0.011**
• Mean ± SD	3.5 ± 3.3	1.6 ± 2.3	
• Median (Min–Max)	3 (0–15)	0 (0–6)	
DASS-21 stress			**0.062**
• Mean ± SD	6.0 ± 4.2	4.0 ± 3.3	
• Median (Min–Max)	5 (0–19)	3.5 (0–10)	
VAS: emotional state			**0.923**
• Mean ± SD	6.0 ± 2.4	6.1 ± 2.2	
• Median (Min–Max)	6 (1–10)	6.5 (1–9)	
VAS: understanding of prognosis			**0.281**
• Mean ± SD	7.9 ± 1.9	8.4 ± 1.8	
• Median (Min–Max)	8 (3–10)	9 (5–10)	
VAS: satisfaction with information			**0.058**
• Mean ± SD	8.4 ± 1.9	7.5 ± 1.9	
• Median (Min–Max)	9 (3–10)	8 (4–10)	

Satisfaction with musical genre			**0.722**
• Mean ± SD	9.3 ± 1.2	9.1 ± 1.7	
• Median (Min–Max)	10 (4–10)	10 (4–10)	

Table S2. Baseline Psychological Characteristics in the Classical Music Group According to Surgical Experience

**Variable**	**Previous surgery (n=56)**	**First surgery (n=17)**	**Mann–Whitney U test (p-value)**
DASS-21 Anxiety	2.6 ± 2.8	3.1 ± 2.9	0.564
DASS-21 Depression	2.6 ± 3.0	3.9 ± 3.1	0.103
DASS-21 Stress	5.1 ± 3.6	6.9 ± 3.8	0.100
VAS: Emotional State (past week)	6.9 ± 1.8	6.3 ± 2.8	0.614
VAS: Understanding of Prognosis	7.9 ± 1.8	7.9 ± 2.5	0.664
VAS: Satisfaction with Information	8.6 ± 1.4	8.3 ± 1.4	0.500
Satisfaction with Musical Genre	8.7 ± 1.9	8.3 ± 2.4	0.577

Table S3. Baseline Psychological Characteristics in the Silence Group According to Surgical Experience

**Variable**	**Previous surgery (n=53)**	**First surgery (n=20)**	**Mann–Whitney U test (p-value)**
DASS-21 anxiety	2.4 ± 3.0	3.6 ± 4.9	0.599
DASS-21 depression	2.5 ± 3.0	2.4 ± 3.7	0.597
DASS-21 stress	4.6 ± 3.4	5.5 ± 4.9	0.681
VAS: emotional state (past week)	7.0 ± 2.3	7.2 ± 1.6	0.910
VAS: understanding of prognosis	8.1 ± 2.2	8.4 ± 1.7	0.653
VAS: satisfaction with information	8.3 ± 2.1	8.1 ± 1.8	0.574
Satisfaction with musical genre	8.5 ± 2.3	8.5 ± 2.5	0.984
